# “Tired of watching customers walk out the door because of the smoke”: a content analysis of media coverage of voluntarily smokefree restaurants and bars

**DOI:** 10.1186/s12889-015-2094-6

**Published:** 2015-08-08

**Authors:** Patricia A. McDaniel, Naphtali Offen, Valerie Yerger, Susan Forsyth, Ruth E. Malone

**Affiliations:** Department of Social and Behavioral Sciences, University of California, San Francisco, 3333 California Street, Suite 455, San Francisco, CA 94118 USA

**Keywords:** Voluntary tobacco control policies, Smokefree public places, Media content analysis

## Abstract

**Background:**

News media are key sources of information regarding tobacco issues, and help set the tobacco control policy agenda. We examined US news coverage of voluntarily smokefree restaurants and bars in locales without mandatory policies to understand how such initiatives are perceived.

**Methods:**

We searched three online media databases (Access World News, Lexis Nexis, and Proquest) for all news items, including opinion pieces, published from 1995 to 2011. We coded retrieved items quantitatively, analyzing the volume, type, provenance, prominence, and content of news coverage.

**Results:**

We found 986 news items, most published in local newspapers. News items conveyed unambiguous support for voluntarily smokefree establishments, regardless of venue. Mandatory policies were also frequently mentioned, and portrayed positively or neutrally. Restaurant items were more likely to mention health-related benefits of going smokefree, with bar items more likely to mention business-related benefits.

**Conclusion:**

Voluntary smokefree rules in bars and restaurants are regarded by news media as reasonable responses to health and business-based concerns about worker and customer exposure to secondhand smoke. As efforts continue to enact comprehensive smokefree policies to protect all in such venues, the media are likely to be supportive partners in the advocacy process, helping to generate public and policymaker support.

## Background

News media are key sources of information about a variety of public health issues, including tobacco, and a high volume of tobacco-related news coverage is associated with negative views of and more accurate beliefs about smoking [1, 2]. The news media also help to advance public health policy, setting the policy agenda by singling out particular issues for discussion [3, 4]. Agenda setting communicates to the public and policymakers the relative importance of various issues based on the amount of media attention devoted to them [4]. It can increase public discourse about an issue and increase the likelihood of a policy response [5].

Researchers have examined media coverage of a range of tobacco topics [5–15]. However, no previous studies have examined the phenomenon of voluntary smokefree policies in restaurants and bars. Voluntary rules restricting smoking in restaurants and bars frequently precede passage of smokefree legislation [16]. While they are by nature less comprehensive and potentially less stable than mandatory policies [17], they may help create public support for legislation by increasing familiarity with and knowledge of the benefits of smokefree public places [16]. However, voluntary measures may stem from different motivations than mandatory policies, which are typically framed as public health interventions [18, 19]. For example, economic considerations, such as reduced cleaning costs or a desire to cater to a majority nonsmoking clientele may motivate restaurant and bar owners to prohibit smoking, rather than concerns about limiting workers’ and patrons’ exposure to a known carcinogen [20]. Similarly, patrons may appreciate smokefree restaurants more for eliminating an unpleasant smell than for protecting their health [18]. Greater knowledge of media coverage of voluntary smokefree measures may provide insight into how to enhance public and policymaker support for comprehensive smokefree legislation, particularly in the case of bars, which many Americans view as venues to which smoking is integral [21]. (There remains a disparity between the number of states with smokefree bar laws (32) versus smokefree restaurant laws (38)) [22].

In this paper, we explore US media coverage of voluntarily smokefree restaurants and bars from 1995-2011. We sought to learn how the media covered this phenomenon in general, including the motivations for and expected benefits of going smokefree voluntarily, and explored whether, and how, media coverage of smokefree restaurants differed from coverage of smokefree bars.

## Methods

We searched three online media databases (Lexis Nexis, Proquest, and Access World News) for news items published between 1995 and 2011 concerning US restaurants or bars that had gone smokefree voluntarily (before the imposition of any local or state clean indoor air laws). The three databases covered 1381 news sources, including 999 local and national newspapers, 11 magazines, 61 newswires, 256 web-only news sources, 53 television network news broadcasts, and National Public Radio. We used a variety of search terms to locate news items, starting with general terms intended to capture all independent or chain restaurants and bars (including taverns and pubs) or restaurant/bar combinations that had voluntarily implemented smokefree indoor air policies (e.g., (voluntar* AND (smoking OR "smoke free")) AND (voluntar* AND (bar OR restaurant OR eatery OR cafe OR dining OR tavern OR pub)). We used retrieved items to identify more specific search terms (e.g., the names of particular restaurants which had gone smokefree). We stopped searching once no new items were found. We included items with nearly-identical content that were published in multiple news outlets in order to understand the reach of media coverage.

We coded news items through a collaborative, iterative process. Using an adaptation of a codebook from an earlier project that examined media coverage of retailers who had voluntarily ended tobacco sales [23], the authors created an initial coding sheet and piloted it on 10 news items.After discussion, we refined and edited the coding sheet and drafted coding instructions. Next, three coders (the second, third, and fourth authors) independently coded an overlapping set of 20 % (n = 201) of the items (chosen with a random number generator), checking in with one another and the first author early in the process to compare results, discuss discrepancies, and refine coding instructions.

We assessed inter-coder reliability of the overlapping sample using Gwet’s AC1 statistic. It is an improvement on the kappa (κ) statistic, which becomes unreliable without sufficient variety in coding [24]. For example, if on one item the correct code is “no” 90 % of the time, the resulting κ has a low value even when inter-rater agreement is high [25–27]. Like the κ statistic, AC1 has a value of 0-1, and can be interpreted in a similar manner. Each variable was tested and variables that did not achieve a value of .60 were not retained. Only one variable was not retained and not used in the analysis. Average inter-coder reliability for all retained variables was 0.833, which has been characterized as “almost perfect” agreement [28, 29].

After confirming inter-coder reliability with the overlapping sample [24], each coder independently coded one-third of the remaining (randomly assigned) news items. We also recoded the items coded early in the process to be consistent with the final version of the codebook. We coded story characteristics (i.e., news source, story type, date, photo, page number, word length, etc.) and content. For the purposes of this paper, we focused our analysis on content related to the overall impression of voluntary smokefree policies, overall customer reaction, health and business-related motivations and outcomes, evidence and authorities cited, and mention and portrayal of mandatory smokefree policies. In determining overall impression and overall customer reaction, we assessed support or not for smokefree policies as reflected in each news item *as a whole*; thus, for example, an item that included one opposition statement and seven statements of support was coded as supportive. We did not conduct significance testing because the items collected were not a random sample, and we are not extrapolating from them. Rather, we report the findings from the entire population of items meeting the search criteria [30].

This study has limitations. The news databases we searched are not comprehensive, although they cover a wide range of national and local newspapers. Our search terms, while comprehensive, may not have been exhaustive; thus, we may have failed to identify and include relevant news items in our study. We also chose to include nearly identical content published by different sources in order to capture the breadth of coverage; as a result, any similar content was coded multiple times. Our results, therefore, reflect *all* coverage that appeared, not *unique* stories.

## Results

### Characteristics of news items and trends over time

We found 986 news items published from 1995-2011 about restaurateurs and/or bar owners who voluntarily prohibited smoking on their premises; the vast majority were local newspaper articles (89.7 %) (daily or weekly newspapers serving a specific city or region, such as the *San Francisco Chronicle*), but sources also included national newspapers (newspapers, such as the *New York Times*, that circulate throughout the US), news wires, magazine articles, and web-based news sites (Table 1). News stories or features comprised the majority of items (71.1 %). Item length ranged from 26 (for brief blurbs) to 3527 words, with a median of 490 words. Most of the coverage concerned smokefree restaurants (65.6 %). 130 items (13.2 %) were duplicates, nearly identical wire service stories published in multiple local newspapers. (We conducted several analyses with duplicates removed, but the results did not differ markedly from those that included duplicates).

**Table 1:** Characteristics of news items on restaurants/bars voluntarily going smokefree, 1995-2011 (N = 986)

Variable	Number	Percent
News source		
Local newspaper	884	89.7
News wire	90	9.1
National newspaper^a^	8	.8
Web-based	3	.3
General audience magazine	1	.1
Geographic region		
West	87	8.8
Midwest	378	38.3
South	259	26.3
Northeast	160	16.2
National^b^	102	10.3
Story type		
News/feature	701	71.1
Blurb^c^	55	5.6
Editorial/op-ed	118	12.0
Letter to the Editor	61	6.2
Column	30	3.0
Press release	21	2.1
Op-ed/letter/column/press release written by health advocate	23	11.0
Prominence (newspapers only; n = 892)		
Front page	119	13.3
First page of section	173	19.4
Photo	214	24.0
Business type		
Restaurant	647	65.6
Bar/restaurant combination	135	13.7
Both restaurants and bars	112	11.4
Bar	66	6.7
Unclear	26	2.6

^a^The *New York Times*, *Wall Street Journal*, *Washington Post*, *Los Angeles Times*, *Christian Science Monitor*, and *USA Today*

^b^News items published in national newspapers, magazines, or on the web, and news items broadcast by National Public Radio or by national television news (CNN, NBC, CBS, FOX, and ABC)

^c^Brief announcement, often included in summaries of current events

**Fig. 1:**
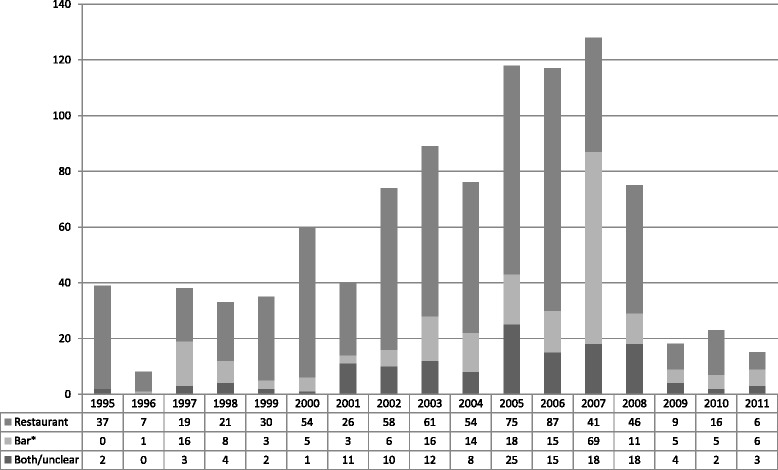
Number of news items about restaurants and bars voluntarily going smokefree, by year, 1995-2011 (N=986). *Includes combined bar/restaurant establishments

The volume of news coverage varied between 1995 and 2011 (Fig. 1). Starting in 2000, there was a trend of increasing coverage that peaked between 2005 and 2007, followed by declining coverage. In every year but two (2007 and 2011), news coverage of voluntarily smokefree restaurants exceeded that of voluntarily smokefree bars.

In newspapers, issues considered editorially important are likely to be given greater prominence – placed on the front page, the front page of a section, or accompanied by a photograph [31]. In our study, among items published in newspapers, 119 (13.3 %) appeared on the front page, 173 (19.4 %) appeared on the first page of a section (other than the section containing the front page), and 214 (24 %) had photos accompanying the articles*.*

### Support for voluntary smoke-free restaurant and bar policies

News coverage was overwhelmingly supportive of these voluntary policies (Table 2). Among all non-opinion news items, the overall impression of restaurant and bar owners’ decision to go smokefree was largely positive (78 %); among editorials, letters to the editor, and columns, nearly all (97.1 %) expressed support for such decisions. For example, one letter writer advocated for voluntary smokefree policies on the grounds that it was good for business, noting that “the first nonsmoking sports bar to open in Ft. Collins, Colo. is still STANDING ROOM ONLY (emphasis in original) even on weeknights after almost two years” [32]. There was also relatively limited mention of any arguments used to oppose voluntary smokefree policies (31.1 %) (e.g., an assumption that the business would lose money or the assertion that alternatives such as ventilation or separate sections were preferable). Customer reaction, when cited, was less overwhelmingly supportive, but nonetheless mostly positive (54.3 %) or mixed (34.2 %), perhaps reflecting the journalistic convention of seeking “both sides” of a story. For example, the manager of a restaurant in southern California described the responses to his newly-adopted smoke-free policy: “A few smokers felt their rights were being trampled”, but “that’s been truly a rare occurrence” [33].

**Table 2:** Content of news items on restaurants/bars going voluntarily smokefree, 1995-2011 (N = 986, except as noted below)

Measure	All venues^a^		Restaurants		Bars		Example
	Total	%	Total	%	Total	%	
Overall impression of smokefree policy conveyed by all non-opinion items (n = 777 all venues; n=530 restaurants; n=131 bars)							
Positive	606	78.0	419	79.1	99	75.6	“Restaurants are finding it profitable and popular to ban smoking entirely.” [53]
Negative	12	1.5	9	1.7	2	1.5	“The owner had been enthusiastic about his smoke-free environment for the night club crowd, but the crowd never came.” [54]
Neutral	159	20.5	102	19.2	30	22.9	“Only a few Ft. Wayne restaurants have voluntarily gone smokefree, and they’ve had differing results.” [55]
Overall impression of smokefree policy in editorials, letters, & columns (n = 209 all venues; n = 117 restaurants; n = 70 bars)							
Positive	203	97.1	113	96.6	70	100.0	“Owners…deserve credit for being the latest Fairbanks restaurants to toss their ashtrays.” [56]
Negative	1	.5	0	0.0	0	0.0	“Most people don’t care about smoke-free establishments.” [57]
Neutral	5	2.4	4	3.4	0	0.0	“…holding his breath to see if his café can survive the no-smoking rule which he initiated.” [58]
Opposition							
Item mentions any opposition to voluntary smokefree policy	307	31.1	193	29.8	55	27.4	“We can’t afford to have it [be smokefree] because we’d lose a lot of business.” [59]
Overall customer reaction reported (n = 444 all venues; n = 297 restaurants; n = 72 bars)							
Positive customer reaction to voluntary smokefree policy	241	54.3	162	54.5	42	58.3	“We just enjoyed the atmosphere. I could breathe…We didn’t smell of smoke.” [60]
Mixed or neutral customer reaction	168	37.8	110	37.0	25	34.7	“A lot of them have boycotted me…But…I have those who are not afraid to come in now.” [61]
Negative customer reaction	35	7.9	25	8.4	5	6.9	“We have had customers refuse to do any more business with us.” [62]
Health-related motivations and outcomes (n = 647 restaurants; n = 201 bars)							
Health cited as reason for implementing voluntary policy	361	36.6	247	38.2	46	22.9	“When secondhand smoke isn’t causing cancer, it is busy irritating the eyes, nose, throat and lungs of nonsmokers.” [56]
Public health advocacy cited as reason for implementing policy	174	17.6	134	20.7	15	7.5	“Smoke Free Mohawk Valley has encouraged numerous restaurants to voluntarily ban smoking.” [63]
Policy considered likely to benefit health^b^	266	27.0	182	28.1	33	16.4	“These businesses have chosen to promote a healthier environment for their patrons and workers.” [64]
Business-related motivations and outcomes (n = 647 restaurants; n = 201 bars)							
Business considerations cited as reason for implementing policy^c^	560	56.8	380	58.7	86	42.8	“It’s not worth it to spend $100,000 to build a smoking section.” [65]
Policy considered likely to benefit business^d^	554	56.2	329	50.9	154	76.6	“The result has been steady sales and more positive feedback.” [66]
Evidence and authorities cited (n = 647 restaurants; n = 201 bars)							
Mention of scientific evidence about tobacco (including health effects)	372	37.7	260	40.2	41	20.4	“[There is] ‘overwhelming scientific evidence’ that secondhand smoke causes heart disease, lung cancer and a list of other illnesses". (Surgeon General Richard Carmona) [67]
Direct quotes from government officials	350	35.5	223	34.5	59	29.4	“This is a public health issue.” (Tucson Councilwoman) [68]
Direct quotes from tobacco control advocacy group representatives	268	27.2	182	28.1	51	25.4	“Smokers don’t quit eating in their favorite restaurant because it is smoke-free. They just quit smoking in it.” (Wisconsin Initiative on Smoking and Health) [69]
Direct quote from restaurant/bar assoc. representative	133	13.5	75	11.6	29	14.4	“We believe it should be a business decision and left to the business owner.” (Wisconsin Restaurant Association) [69]
Direct quote from tobacco industry representative	34	3.4	30	4.6	0	0.0	“We believe adults should be able to patronize establishments that permit smoking if they choose to do so.” (RJR spokesperson) [70]
Mandatory policies mentioned (n = 695 all venues; n = 404 restaurants; n = 168 bars)							
Positive portrayal	230	33.1	126	31.2	74	44.0	“It’ll probably help people quit smoking.” [71]
Negative portrayal	79	11.4	55	13.6	11	6.5	“Legislation to force business owners to convert to nonsmoking is neither appropriate nor required.” [72]
Neutral or mixed portrayal	386	55.5	223	55.2	83	49.4	“A bill to enact a statewide smoking ban failed in the Alabama Legislature…Do than and…other cities in the state, however, have enacted local ordinances severely limiting smoking.” [73]

^a^All venues include 112 items focused on *both* restaurants and bars, and 26 items whose focus was unclear

^b^Health benefits included less smoking in general, encouraging kids not to smoke, improving health, and “benefiting people”

^c^Business considerations included financial motivations, a desire to improve the business’s image, accommodating nonsmoking customers, protecting property, anticipating a mandatory law, following industry trends, and limited space to accommodate smokers

^d^Business benefits included a better image, a gain or no change in income/patronage, a cleaner, fresher smell, and a “general benefit”

### Health and business-related motivations and outcomes

News items referenced a variety of health and/or business-focused reasons for and likely consequences of restaurants and bars going smokefree voluntarily. One of the most common reasons given for implementing voluntary smokefree policies was to promote health (e.g., of customers or employees) (36.6 %) (Table 2); however, taken together, business reasons, such as accommodating nonsmoking customers or reducing cleaning costs, were more commonly cited (56.8 %). For example, a restaurant owner in Michigan reportedly “put an end to lighting up” because “he was tired of watching customers walk out the door because of the smoke” [34]. A restaurant manager in North Dakota noted that going smokefree would save on such regular expenses as cleaning “the blackened filters of the ventilation system” [35]. Similarly, when discussing the potential positive impacts of going smokefree, news items more often cited benefits that would accrue to the business, such as an improved image or a gain (or at least no change) in patronage (56.2 %), rather than public health benefits such as a reduction in smoking or improved health (27.0 %). For example, an Idaho bar owner said that “voluntarily making his bars smoke-free has been a boon for business. … [It] has meant lower labor costs, no clogged urinals and no cigarette burns on the furniture” [36].

### Evidence and sources quoted

In print articles, direct quotes are more influential on readers’ impressions of issues than paraphrased quotes [37]; thus, we coded sources in news items who were directly quoted. Government officials (including elected officials and heads of public health departments) and tobacco control advocates, while not quoted regularly, were quoted more often than representatives of the tobacco industry and its allies, restaurant and bar associations (Table 2) [38, 39]. Elected officials were often quoted debating the pros and cons of legislation mandating smokefree restaurants or bars. For example, one North Dakota State Representative discussed constituent input about smokefree legislation, noting that she had “not had the type of push for bars (to ban smoking) that we got for the restaurants from the public” [40]. Tobacco control advocates typically praised businesses for going smokefree. When two popular chain restaurants went smokefree, John Kirkwood, the President and CEO of the American Lung Association, offered his congratulations in a 2005 wire service article, noting that “[t]his shows that they are concerned about the health of their employees and customers” [41].

Nearly 40 % of articles referenced scientific evidence about tobacco, including its deadly disease effects, to help provide context for business owners’ decisions to prohibit smoking in their establishments (Table 2). For example, an article featuring a restaurant with a bar that went smokefree in upstate New York noted that “Statistics show that for every 8 smokers killed by tobacco, 1 nonsmoker also dies” [42]. We examined whether mention of scientific evidence about tobacco increased after the 2006 publication of the Surgeon General’s report on secondhand smoke [43]; we found no such increase.

### Coverage of mandatory policies

With a steady rise in the number of smokefree laws over the period of study [17], it was unsurprising that a majority of news items (70.5 %) referred to one or more laws governing smoking (typically, but not exclusively, in restaurants or bars) (Table 2). Given the high level of support for voluntary rules in news items, we expected support for mandatory policies to be lower; while this was the case, portrayals of mandatory policies were still more likely to be mixed or neutral (55.5 %) or even positive (33.1 %) than entirely negative (11.4 %). A common criticism of such policies was that they represented governmental inconsistency *and* infringed on business owners’ rights (mentioned in 33.8 % of stories that cited mandatory policies). One bar owner in Corpus Christi, Texas who voluntarily imposed a smoke-free rule on his premises exemplified this sentiment: “I’m not sure the government should tell people what they can and cannot do. As a business owner, tobacco is a legal drug. If it’s so bad, why don’t they ban it altogether?” [44].

### Restaurants versus bars

Given that smokefree bars appear to be less widely accepted by the public than smokefree restaurants [21], we compared coverage of the two venues to assess whether this was reflected in news coverage. (We combined news items concerning bars and restaurant/bar combinations into the same category (bars) because it seemed reasonable to assume that an establishment with both a restaurant and bar was likely to share many features of a stand-alone bar and to elicit a similar public response when going smokefree.) First, we examined characteristics of news items for each venue. The only notable differences concerned the type of news story and media region. Editorials and news items were more common among bar-related items (61, 30.3 %) than restaurant-related items (49, 7.6 %), and, among all regions, the Midwest claimed nearly half of all bar-related items (100, 49.8 %).

Next, we examined the slant of coverage. Both opinion and non-opinion news items were overwhelmingly supportive, regardless of venue (Table 2). Reported customer reaction was also quite similar for both bars and restaurants, with the majority clustered in the positive or mixed categories rather than purely negative. One notable difference in the slant of coverage of the two venues concerned mandatory smokefree policies for restaurants or bars. Bar-related items were more likely to mention such policies (168, 83.6 % versus 404, 62.4 %), and to portray them favorably (44.0 % versus 31.2 %; Table 2). For example, in an article that reported on a smoke-free bar in Bowling Green, Kentucky, a tobacco control advocate noted that “she’s found that more people in the city would favor a citywide smoking ban than not” [45].

We also explored venue-related differences in motivations for and expected benefits of going smokefree. Overall, restaurant items were more likely than bar items to cite *any* reason for going smokefree, including health (38.2 % versus 22.9 %), public health advocacy (20.7 % versus 7.5 %), or business considerations (58.7 % versus 42.8 %) (Table 2). Restaurant items were also more likely to mention health-related benefits of going smokefree (28.1 % versus 16.4 %), while bar items were more likely to mention business-related benefits (76.6 % versus 50.9 %) (Table 2). For example, when Mike Scanlon, owner of Applebee’s and Johnny Carino’s chains, announced that all of his restaurants had gone smoke-free, he explained, “We’re not killing employees anymore” [46]. A Wyoming bar owner stressed the economic benefit of going smokefree: “It actually has been going incredibly well. Our business is up since we did it” [47].

Finally, we examined the evidence and authorities cited in restaurant versus bar items. While the likelihood of citing various authorities did not differ markedly, restaurant-related items were twice as likely to mention scientific evidence about tobacco, including its disease effects (40.2 % versus 20.4 %) (Table 2).

## Discussion

Restaurant and bar owners’ decisions to voluntarily go smokefree in their establishments were newsworthy events, reported on frequently over the 17-year period of the study (although with a decline in coverage starting in 2008). They were primarily the subject of local media attention, most likely because the majority of businesses that received coverage were local, independent restaurants or bars; a change in their smoking policy was likely to be of interest to local residents. Some of the coverage also occupied prominent positions in newspapers (e.g., on the front page or the first page of a section) or had an accompanying photograph, likely to draw readers’ attention, suggesting that the topic was considered not just of interest but likely to be important to community members.

Voluntarily smokefree restaurants and bars were a topic more common in the media in the Midwest and the south, regions that have been slower to introduce smokefree restaurant and bar laws [48] and are thus perhaps more reliant on voluntary measures. However, despite regular media attention over the period, coverage peaked in the mid-2000s, possibly because interest waned over time, as smokefree restaurants and bars became less novel (and hence less newsworthy) [49], or because fewer voluntary smokefree rules were introduced after 2007, replaced by smokefree legislation or reaching a saturation point among business owners.

Surprisingly, on several measures, news items conveyed unambiguous support for voluntarily smokefree establishments, regardless of venue. Public ambivalence about smokefree bars [21] was clearly not reflected in the media. It did not appear to be the case that this support hinged on the voluntary nature of the policy, as bar-related items also frequently mentioned mandatory policies, and typically portrayed them in a positive or neutral fashion. Instead, the high level of support for voluntarily smokefree bars in media coverage may have been explained by the inclusion of pragmatic business-related reasons for and expected benefits of going smokefree, coupled with changes in social norms about tobacco use more generally.

There were, however, some notable venue-specific differences in coverage. Smokefree bars generated more editorials and op-eds than smokefree restaurants, suggesting that editors regarded smokefree bars as more novel or worthy of more interpretation for readers [8], possibly because they were considered more likely than smokefree restaurants to generate controversy. Comparing coverage of the two venues also revealed some important differences in health and business motivations for and outcomes of going smokefree, with health considerations playing a larger role in restaurant-related items. Restaurant items were also twice as likely to cite scientific evidence about tobacco, including its negative disease effects. It may be more normative for restaurant owners to cite health concerns as reasons for going smokefree. Restaurants serve adults and children, and at least some promise healthful products; by contrast, bars are adults-only venues already associated with a (potentially) hazardous behavior (drinking).

## Conclusion

Media coverage not only educates readers about the salience of particular issues, but also helps them interpret and respond to those issues [1, 4, 50–52]. The American news coverage we analyzed conveyed to readers both that voluntarily smokefree bars and restaurants were an important issue, and that they were a reasonable response to health and business-based concerns about worker and customer exposure to secondhand smoke. In addition, most media coverage did not imply that voluntary policies were preferable to mandatory policies. As efforts continue in the U.S. to enact comprehensive smokefree policies to protect all workers and customers in such venues, the media are likely to be supportive partners in the advocacy process, helping to generate public and policymaker support.
